# Polymerase Chain Reaction-Confirmed Lyme Neuroborreliosis Masked by Pseudomonas Mastoiditis in a Mexican Traveler: A Case Report

**DOI:** 10.7759/cureus.95596

**Published:** 2025-10-28

**Authors:** César A Vega-López, Ximena Barrientos, Sandra Rivera, Marité Palma-Díaz, Santiago Obregón-Rosas

**Affiliations:** 1 Internal Medicine, Hospital Angeles Pedregal, Mexico City, MEX; 2 Faculty of Medicine, La Salle University, Mexico City, MEX; 3 Subdirectorate of Teaching Coordination, Instituto Nacional de Cardiología - Ignacio Chávez, Mexico City, MEX

**Keywords:** central nervous system infections, coinfection, delayed diagnosis, facial nerve palsy, lyme disease, mastoid abscess, neuroborreliosis, pcr diagnosis, pseudomonas aeruginosa, travel medicine

## Abstract

Lyme borreliosis is seldom suspected in Mexico, yet vectors and human cases have been documented, and climate‑driven range expansion is predicted. We report a 67‑year‑old Mexican woman who, four months after suboptimally treated right‑sided otitis media, developed ipsilateral facial palsy, followed by episodes of fluctuating consciousness and cognitive impairment, suggestive of encephalopathy and rapidly progressive paraparesis. Imaging demonstrated destructive mastoiditis with a post‑sternocleidomastoid abscess from which *Pseudomonas aeruginosa* was cultured. Despite adequate surgical drainage and broad-spectrum antibiotic therapy, the patient’s neurological status continued to deteriorate. Although cerebrospinal fluid (CSF) parameters remained within normal limits, intravenous ceftriaxone led to rapid stabilization of cognitive function, prompting reevaluation for a possible tick-borne etiology. Retrospective history revealed travel to San Antonio, Texas, 24 months earlier. *Borrelia burgdorferi* DNA was detected in CSF by polymerase chain reaction (PCR), and serum IgG was positive, fulfilling the guideline criteria for definite Lyme neuroborreliosis. A six‑week course of ceftriaxone, followed by oral doxycycline, led to near‑complete neurological recovery, except for persistent facial paresis. This case underscores three teaching points: (1) Lyme neuroborreliosis should enter the differential diagnosis of subacute cranial neuropathy and radiculomyelitis even in so‑called non‑endemic regions when travel or ecological change is plausible; (2) CSF may be normal and PCR sensitivity is low, yet a positive result is highly specific and can be decisive; and (3) superimposed nosocomial infections, here a pseudomonal mastoid abscess, may obscure the underlying vector‑borne disease and delay targeted therapy. Heightened clinical vigilance and adherence to diagnostic algorithms are essential to prevent irreversible disability as the geographic footprint of *Ixodes* ticks widens.

## Introduction

Lyme borreliosis is the most common tick‑borne zoonosis in the Northern Hemisphere, and insurance‑based modelling now places the annual number of patients diagnosed and treated in the United States at roughly 476,000, far exceeding routine surveillance tallies and illustrating the large hidden burden of disease [1]. The infection is caused by the spirochete *Borrelia burgdorferi *sensu lato and transmitted to humans by *Ixodes *ticks; in North America, *B. burgdorferi *sensu stricto predominates, whereas *B. garinii *and *B. afzelii *prevail in Europe, a geographic variability that shapes clinical phenotypes [2].

Beyond its well-recognized vector dynamics, *B. burgdorferi *expresses several virulence factors that enable persistent infection, including outer surface proteins (Osps) that mediate adhesion to host tissues and the variable major protein-like sequence (VlsE) system that promotes antigenic variation and immune evasion. These mechanisms facilitate hematogenous dissemination to the nervous system, joints, and skin, producing a wide clinical spectrum. Neurological involvement most commonly manifests as painful meningoradiculitis, cranial neuropathy - particularly facial nerve palsy - or lymphocytic meningitis, collectively referred to as Lyme neuroborreliosis [2].

Although Mexico has traditionally been considered a region of low endemicity, the first molecularly confirmed autochthonous case, detection of *B. burgdorferi *DNA in local ticks, and a cumulative series of 398 human reports, suggest under-recognition and the possibility of future expansion of the vector’s range [3]. The most comprehensive seroepidemiological survey to date demonstrated antibody positivity in 3.43% of residents of Mexico City and up to 6.2% in northern states; within that cohort, neurological manifestations were prominent: 27.4% presented polyradiculoneuropathy, 28.5% facial palsy, 22.6% encephalomyelitis, and 20.8% other neuropathies [4]. Neurological involvement develops in about 10-15% of untreated infections and represents the second most frequent disseminated manifestation in contemporary European cohorts, underscoring its importance for clinicians practicing inside and outside endemic areas [3]. We describe a 67‑year‑old Mexican woman who developed neuroborreliosis months after traveling to Texas and whose course was complicated by a mastoid abscess and bacteremia due to *Pseudomonas aeruginosa*, circumstances that delayed recognition of the underlying tick‑borne illness, illustrating how coinfection can confound diagnostic reasoning.

## Case presentation

A 67-year-old female with a medical history of type 2 diabetes mellitus, high blood pressure, Parkinson’s disease, and hypothyroidism presented to the emergency room with right-sided otalgia and hearing loss.

At initial presentation, approximately six weeks after prior discharge, the patient reported severe right-sided otalgia and retroauricular headache radiating to the temporal and parietal regions, rated 10/10 on the numeric pain scale, persistent and nocturnally exacerbated. She remained afebrile throughout the course and denied vertigo or ear discharge. Her symptoms progressively worsened with ipsilateral facial palsy, dysphagia to liquids, and left-sided weakness.

Following a diagnosis of right-sided otitis media, initial inpatient therapy consisted of empirical intravenous ceftriaxone plus levofloxacin. This regimen was selected to provide broad coverage for common otitis pathogens and potential Gram-negative organisms and resulted in partial symptom relief.

**Figure 1:**
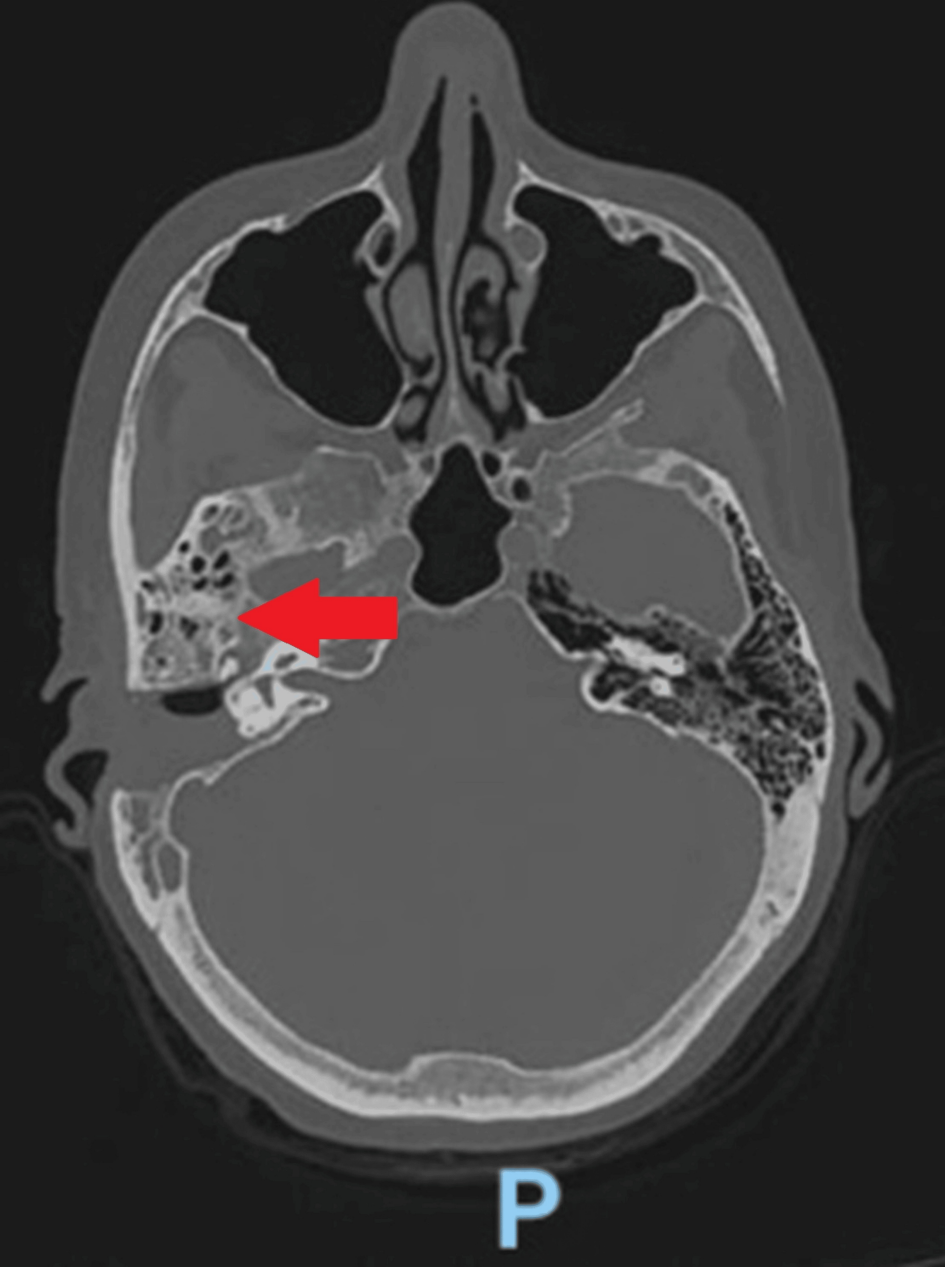
CT scan of the head and ear. The study shows an image of right-sided otomastoiditis with associated morphological changes in the posterior mastoid air cells and the mastoid tip (red arrow).

Four months later, she developed right-sided facial paralysis, hyperesthesia, and pain localized to the same region. Physical examination revealed right cranial nerve VII palsy with localized tenderness. Contrast-enhanced CT of the ear and neck showed severe mastoiditis with bony destruction of the mastoid tip and extension into the neck, forming a post-sternocleidomastoid abscess (Figure 1). The patient underwent surgical drainage with facial nerve decompression. *P. aeruginosa* was isolated from the abscess culture, and she was discharged on intravenous meropenem and vancomycin.

Despite treatment, she continued to experience retroauricular headache radiating to the temporal and cervical areas, as well as persistent ipsilateral facial paralysis. Two months later, she developed bilateral lower limb weakness, preventing ambulation and fluctuating levels of consciousness, with episodes of somnolence. Additionally, she presented with dysphagia to liquids and hypoesthesia in the right mandibular region.

On re-admission, she was somnolent, with persistent right facial hemiparesis and marked lower limb weakness, more prominent on the left (1/5 on the Daniels muscle strength scale), accompanied by paresthesia. Mental status fluctuated between somnolence, disorientation, and episodes of agitated or uncooperative behavior consistent with encephalopathy.

**Figure 2:**
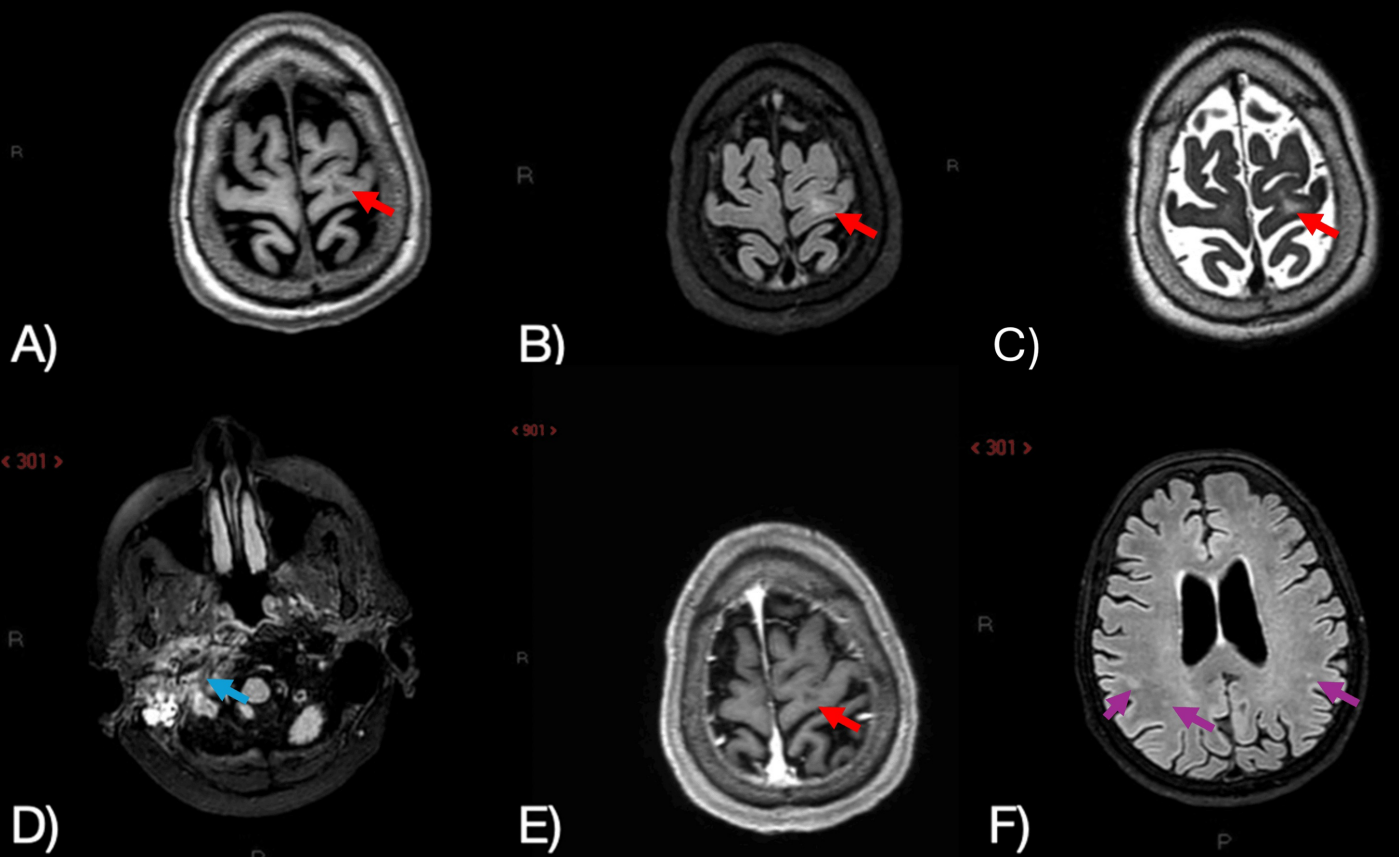
Magnetic resonance imaging (MRI) of the patient’s brain. Several hyperintense lesions are seen in the sub‑cortical white matter, the largest in the left pre‑central gyrus. Such findings are common in age‑related microangiopathy; nonetheless, lesions caused by *Borrelia *infection cannot be ruled out, especially because no contrast enhancement is present. A) Axial unenhanced T1‑weighted sequence: hypointense focus in the dorsal left pre‑central gyrus (red arrow). B) Axial fluid-attenuated inversion recovery (FLAIR, T2‑weighted) sequence: hyperintense focus in the dorsal left pre‑central gyrus (red arrow). C) Axial T2‑weighted sequence: hyperintense focus in the dorsal left pre‑central gyrus (red arrow). D) Axial FLAIR sequence: inflammatory changes in the infratemporal fossa with involvement of the mastoid air cells and the Eustachian tube (blue arrow). E) Axial post‑gadolinium T1‑weighted sequence: no enhancement of the left pre‑central lesion (red arrow). F) Axial FLAIR sequence: scattered hyperintense lesions in the bilateral parietal sub‑cortical white matter (purple arrows); ventricles and subarachnoid spaces are enlarged.

**Figure 3:**
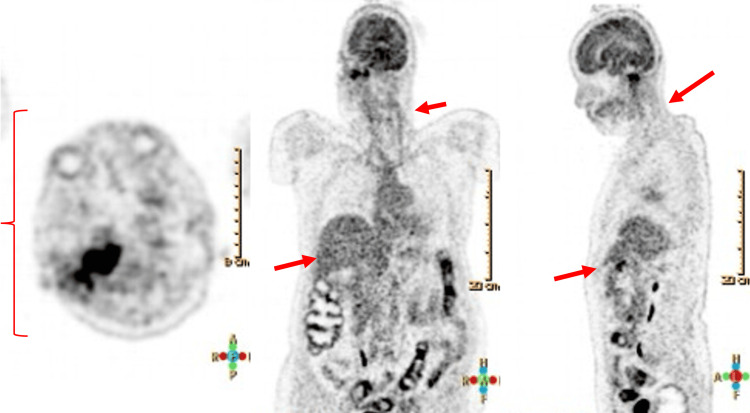
Whole-body PET-CT scan in the evaluation of suspected lymphoma. Maximum intensity projection images from a whole-body PET-CT scan, shown in axial (left), coronal (middle), and sagittal (right) planes. The study was performed to evaluate for an occult neoplastic process given the patient’s subacute neurological decline and mastoid abscess. No areas of abnormal FDG uptake were observed, effectively ruling out systemic lymphoma or other metabolically active malignancies. Red arrows highlight key anatomical regions (brain, neck, and abdomen) assessed during the scan, all of which showed normal metabolic activity.

Due to suspected neuroinfection, lumbar puncture was performed, showing normal cerebrospinal fluid (CSF) parameters (an atypical finding in confirmed cases) (Table 1), with negative cultures and multiplex PCR (*FilmArray*™) (Table 2). Brain MRI revealed a hyperintense area in the mastoid and adjacent structures (Figure 2). PET-CT was performed to rule out lymphoma; results were unremarkable (Figure 3).

**Table 1:** Cerebrospinal fluid analysis. The asterisk indicates the only out-of-range parameter (erythrocytes).

Parameter	Results	Reference Range	Units
Color	Colorless	Colorless/Light yellow	-
Appearance	Transparent	Transparent	-
Coagulum	Absent	Absent	-
Glucose	173.0	50.0-80.0	mg/dL
Total Proteins	41.9	15.0-80.0	mg/dL
Chloride	128	118-130	mmol/L
Leukocytes	0.0	0.0-10.0	Cells/µL
Erythrocytes	20.0*	0.0-10.0	Cells/µL
Lymphocytes	0.0	-	%
Monocytes	0.0	-	%
Neutrophils	0.0	-	%
Macrophages	0.0	-	%
Adenosine Deaminase in CSF	1.12	0.9	U/L
Gram Stain	No microorganisms observed	-	-
Ziehl-Neelsen Stain	Negative	-	-
General Bacterial Culture	No growth	-	-
Fungal Culture	No growth	-	-
KOH Test	Negative	-	-
India Ink Stain (for *Cryptococcus*)	Negative	-	-

**Table 2:** Molecular panel for meningitis/encephalitis (PCR multiplex). PCR: polymerase chain reaction

Pathogen Category	Pathogen Name	Result
Bacteria	*E. coli *K1	Not detected
	H. influenzae	Not detected
	L. monocytogenes	Not detected
	N. meningitidis	Not detected
	S. agalactiae	Not detected
	S. pneumoniae	Not detected
Viruses	Cytomegalovirus	Not detected
	Enterovirus	Not detected
	Herpes Simplex Virus Type 1	Not detected
	Herpes Simplex Virus Type 2	Not detected
	Human Herpesvirus 6	Not detected
	Human Parechovirus	Not detected
	Varicella-Zoster Virus	Not detected
Fungi	Cryptococcus neoformans/gattii	Not detected

Empirical treatment with high-dose intravenous ceftriaxone (e.g., 2 g/day) was initiated. Although the neurological exam remained initially unchanged, relatives noted improved mental status stability. Upon further questioning, the patient’s family reported travel to San Antonio, Texas, in 2022. Based on the patient’s clinical improvement with ceftriaxone, combined with a history of travel to an endemic region within the past 36 months, PCR testing for *B. burgdorferi* was performed and returned positive.

A diagnosis of neuroborreliosis with concomitant *P. aeruginosa *infection was established. The patient showed progressive neurological improvement: she became alert, cooperative, and oriented, with gradually improving lower limb strength. However, the right facial paralysis persisted. She was discharged on a regimen of high-dose intravenous ceftriaxone, followed by oral doxycycline.

Eight weeks post-discharge, the patient demonstrated generalized clinical improvement. She was oriented, cognitively intact, and physically independent, with no motor or sensory deficits in her limbs. Right facial paresis persisted, but laboratory markers of inflammation had decreased, and no leukocytosis was noted.

## Discussion

The patient’s presentation with persistent unilateral facial palsy, fluctuating encephalopathy, and subacute paraparesis reflects the typical spectrum of Lyme neuroborreliosis, where painful meningoradiculitis, lymphocytic meningitis, and cranial‑nerve involvement form the clinical triad; the genospecies distribution helps explain why cranial neuropathy is especially common in American cases dominated by *B. burgdorferi* sensu stricto [2]. Diagnosis in non‑endemic settings is challenging because classic early clues such as erythema migrans are often absent or overlooked; in our patient, the decisive elements were a positive PCR for *B. burgdorferi* in CSF, obtained despite the well‑known sensitivity of <30%, and systemic IgG seropositivity, interpretations that align with guideline recommendations requiring convergent clinical, serologic and, when possible, direct evidence of infection [5,6]. Current Infectious Diseases Society of America (IDSA)-American Academy of Neurology (AAN)-American College of Rheumatology (ACR) guidance endorses ceftriaxone 2 g intravenously once daily or doxycycline 100 mg orally every 12 hours for 14-21 days; a multicenter non‑inferiority trial confirmed the equivalence of oral doxycycline and intravenous ceftriaxone for early neuroborreliosis, but parenteral therapy remains the preferred option when central‑nervous‑system parenchyma is involved or when additional hospital‑acquired pathogens require broad coverage [5,7].

The patient’s comorbidities likely contributed to both susceptibility and clinical course. Diabetes mellitus is known to increase the risk of complicated otitis and invasive bacterial infections, delay wound healing, and favor colonization by Gram-negative organisms, such as *Pseudomonas *species. Advanced age and immunosenescence may further impair pathogen clearance and modify clinical presentation, leading to atypical or more severe disease. Parkinson’s disease may increase vulnerability to complications related to dysphagia or impaired airway protection and complicate rehabilitation, while polypharmacy in elderly patients raises concerns about drug-drug interactions and adverse effects. Although no major antibiotic-related adverse events were documented in the record, awareness of these interactions and vigilant monitoring are warranted when treating similar patients.

The initial choice of intravenous ceftriaxone plus levofloxacin was empirical, aiming to cover typical otitis pathogens and potential Gram-negative organisms in a severe presentation. Culture-directed therapy was instituted after isolation of *P. aeruginosa*. No clinically significant antibiotic-related adverse events were recorded in the medical chart. Nonetheless, clinicians should be mindful of potential fluoroquinolone-associated CNS effects, QT-prolongation risk, tendinopathy, and possible interactions with antiparkinsonian regimens; careful review of concomitant medications and monitoring are advisable in elderly, polymedicated patients.

In our patient, ceftriaxone was maintained both for neuroborreliosis and as part of combination therapy with a carbapenem directed at *P. aeruginosa*, a coinfection seldom reported in Lyme disease but documented in a recent case of Lyme carditis complicated by pseudomonal pneumonia, illustrating how opportunistic bacteria can obscure or prolong the clinical course [8]. Her gradual neurological recovery following dual antimicrobial treatment supports evidence that timely, guideline‑concordant therapy prevents permanent sequelae, although residual facial weakness may persist despite microbiological cure.

This diagnostic delay illustrates how limited clinical familiarity in low-endemic regions may hinder early recognition of Lyme disease. Incorporating travel history and ecological awareness into diagnostic algorithms could improve the timely identification of neuroborreliosis.

## Conclusions

This case exemplifies the diagnostic difficulties posed by Lyme neuroborreliosis in regions regarded as non‑endemic and highlights the necessity of integrating travel history, subtle epidemiologic clues, and comprehensive laboratory testing when evaluating subacute cranial neuropathies or radiculomyelitis. A high index of suspicion, adherence to serologic algorithms, and appreciation of the limited but highly specific role of PCR in CSF can expedite recognition and treatment. Coinfections unrelated to the tick bite, such as invasive *P. aeruginosa* disease in an elderly host with comorbidities, may further confound the picture and demand parallel antimicrobial strategies. As climate change and global mobility extend the habitat of *Ixodes *ticks, clinicians practicing outside traditional endemic zones must remain vigilant for atypical presentations of vector‑borne infections to avoid delayed diagnosis and irreversible neurological disability.

## References

[1] (2025). Lyme disease surveillance and data. https://www.cdc.gov/lyme/data-research/facts-stats/index.html.

[2] Marques AR, Strle F, Wormser GP (2025). Comparison of Lyme disease in the United States and Europe. Emerg Infect Dis.

[3] Colunga-Salas P, Sánchez-Montes S, Volkow P, Ruíz-Remigio A, Becker I (2020). Lyme disease and relapsing fever in Mexico: an overview of human and wildlife infections. PLoS One.

[4] Gordillo-Pérez G, Torres J, Solórzano-Santos F, Garduño-Bautista V, Tapia-Conyer R, Onofre Muñoz M (2003). [Seroepidemiologic survey of Lyme borreliosis in Mexico City and the Northeast region of the country]. Salud Pública Méx.

[5] Lantos PM, Rumbaugh J, Bockenstedt LK (2021). Clinical practice guidelines by the Infectious Diseases Society of America (IDSA), American Academy of Neurology (AAN), and American College of Rheumatology (ACR): 2020 guidelines for the prevention, diagnosis, and treatment of Lyme disease. Arthritis Care Res (Hoboken).

[6] Otto F, Wipfler P, Hitzl W, Preisel M, Harrer A, Pilz G (2024). Cerebrospinal fluid cytology in Lyme neuroborreliosis revisited—role of neutrophilic granulocytes: a retrospective single-center study. J Clin Med.

[7] Ljøstad U, Skogvoll E, Eikeland R, Midgard R, Skarpaas T, Berg A, Mygland A (2008). Oral doxycycline versus intravenous ceftriaxone for European Lyme neuroborreliosis: a multicentre, non-inferiority, double-blind, randomised trial. Lancet Neurol.

[8] Vachss D, Yusuf Y, Bobrow D, Geraghty P (2023). Lyme carditis accompanied with pseudomonal pneumonia: a case report. Am J Med Case Rep.

